# Exploring the mediating role of virtual environment loneliness in the link between interpersonal relationship styles and social anxiety

**DOI:** 10.3389/fpsyg.2025.1603001

**Published:** 2025-08-20

**Authors:** Mustafa Batuhan Kurtoğlu, Kübra Sezer Katar

**Affiliations:** ^1^Department of Psychology, Faculty of Economics, Administrative and Social Sciences, Hasan Kalyoncu University, Gaziantep, Türkiye; ^2^Alcohol and Drug Treatment and Education Center, Department of Psychiatry, Ankara Training and Research Hospital, University of Health Sciences, Ankara, Türkiye

**Keywords:** inhibitory relationship, nurturing relationship, social anxiety, social interaction, virtual loneliness

## Abstract

**Objectives:**

As technology has transformed social interactions, the study investigates whether virtual communication adequately fulfills individuals’ social connection needs. This study explores the relationship between social anxiety, virtual environment loneliness, and interpersonal relationship styles among a diverse sample of 400 participants.

**Methods:**

The study included 400 participants (*N* = 400), the majority of whom were female (*N* = 254, 63.5%) and had at least a bachelor’s degree (*N* = 261; 65%). Participants completed the Environment Loneliness Scale, the Liebowitz Social Anxiety Scale, and Interpersonal Relationships Scale. Multi-mediation analysis was run to assess the potential mediating role of virtual loneliness.

**Results:**

Results showed that an inhibitory relationship style was positively correlated with social anxiety (*r* = 0.30, *p* < 0.01), while a nurturing relationship style was negatively correlated (*r* = −0.21, *p* < 0.01). Mediation analyses revealed that a subdimension of virtual environment loneliness, called virtual sharing, significantly mediated the relationship between inhibitory relationship style and social anxiety. However, no significant indirect effect was observed between nurturing relationship style and social anxiety.

**Conclusion:**

In conclusion, the current study provides insight regarding the relationship between interpersonal relationship styles and social anxiety by emphasizing the role of virtual sharing in this association. Successfully managing social interactions is essential for improving individuals’ psychological well-being. Future studies should further investigate these relationships to optimize interventions for individuals struggling with social anxiety.

## 1 Introduction

Social anxiety is a prevalent psychological condition characterized by an intense fear of social interactions and situations in which individuals anticipate being scrutinized or negatively evaluated by others, restricting their chances of forming meaningful social ties (Morrison and Heimberg, 2013). This condition manifests in various forms, including the avoidance of social gatherings and communication with others, excessive self-consciousness, and physiological responses such as sweating or trembling (Steinert et al., 2013). It adversely impacts interpersonal relationships, disrupts academic, occupational life and daily functioning (Fehm et al., 2008), even may result in other psychiatric disorders (Copeland et al., 2011).

Overall, social anxiety and related disorders are highly prevalent in the society, and mild to extreme levels of social anxiety make people experience difficulty in social situations, retrieve them from having meaningful social interactions, experience adverse physical and psychological symptoms (Alomari et al., 2022). Given its widespread occurrence and detrimental effects on human functioning, examining the underlying mechanisms that contribute to social anxiety remains a crucial interest in psychological research. Within this scope, the current study suggested examining social anxiety from the lenses of interpersonal relationship styles and experience of loneliness in virtual environment.

### 1.1 Interpersonal relationship styles

While there is no definitive pathway to developing social anxiety disorder, challenges in interpersonal relationships can be a significant factor contributing to its onset (Derin et al., 2022). People have a fundamental psychological need to be related to others, which is directly linked to their psychological health (Self-Determination Theory; SDT; Deci and Ryan, 2013). That is why they need to build and maintain meaningful social relations to sustain their well-being and health (Fotiadis et al., 2019; Arslan, 2024). However, the nature of social interactions and how they are expressed can vary significantly from person to person. Perception of the self and others, the way of communication with or expression of the self to others are referred as interpersonal relationships style (Daffern et al., 2010). There are various ways of classifying interpersonal relationships styles. However, to ensure cultural sensitivity in Turkish context, classification framework proposed by Şahin and colleagues was chosen for the present study (Şahin and Durak, 1994). According to their model also see (Hasta and Güler, 2013), interpersonal relationship styles can be either nurturing/positive (characterized by openness and respect) or inhibitory/negative (dismissive, closed, and egocentric way of social interactions).

While nurturing relationships promote mutual understanding and cooperation, inhibitory styles create challenges in maintaining healthy interpersonal interactions (Grenwald and Yıldırımoğlu, 1999), which may lead to undesired outcomes. To illustrate, unlike nurturing relationship style, holding an inhibitory relationship style found to be predictive of loneliness (Şahin and Durak, 1994). Moreover, the nurturing relationship style has been linked to positive psychosocial outcomes, such as increased subjective well-being (Doğan and Sapmaz, 2012) and greater marital satisfaction (Kasapoğlu et al., 2017). In contrast, the inhibitory relationship style has been predominantly associated with negative factors, including attachment anxiety (Kaya et al., 2023) and anxiety disorders (Şahin et al., 2011a), often leading to adverse outcomes such as hopelessness and suicidal tendencies (Arsel and Batigün, 2011). Although there is an increasing interest in psychological correlates of relationship styles, there is a lack of research on interpersonal relationship styles and social anxiety relationship (Manning et al., 2017).

### 1.2 Virtual environment loneliness

Social interactions do not always occur in face-to-face physical environments. With the advancement of technology, the way individuals communicate and socialize has rapidly changed. Face-to-face communication has largely been replaced by messaging, voice or video calls, gaming, and social media interactions (Smith and Anderson, 2018). Particularly, social media usage presents itself as a significant phenomenon affecting daily life, with direct implications for our lives (Amichai-Hamburger and Ben-Artzi, 2003). While interactions and communications in virtual environments offer opportunities similar to those in real life, they also present limitless possibilities. However, it is important to understand whether online social interactions adequately meet individuals’ needs for social connection (Towner et al., 2022). The literature reveals conflicting results regarding what online interactions offer. Yao and Zhong (2014) discovered that as social interactions decreased, online interactions increased. Furthermore, they noted that as online addiction intensified, individuals became increasingly isolated (Yao and Zhong, 2014). Additionally, some social interactions may involve cyberbullying and encourage problematic behaviors such as anorexia or self-harm (Margherita and Gargiulo, 2018; Kowalski et al., 2019; Cosma et al., 2020), while some online support groups can be beneficial for individuals with various conditions, such as depression (Breuer and Barker, 2015). A study conducted with university students found that the perceived usefulness of online support groups was positively related to social support when participants reported moderate to high levels of social anxiety (Ruppel and McKinley, 2015).

Overall, the role of virtual interactions in interpersonal relationships and their mental health outcomes appear to be debatable. Individuals who prefer spending excessive time in virtual environments and struggle with physical presence in social settings may gradually detach from concepts such as time and responsibility. However, this situation, which drives individuals to social isolation, can also lead to various problems in the virtual world. Virtual loneliness is a concept that has emerged in recent times with the exponential increase in internet usage, which may lead to psychological and physical distress (Korkmaz et al., 2014). Nevertheless, in the virtual world, a person may experience loneliness without encountering psychological distress or problems in interpersonal relationships (Moretta and Buodo, 2020). Paradoxically, due to the opportunities it provides, the virtual world may be preferred even though individuals experience loneliness (Morahan-Martin and Schumacher, 2003). As is well known, social anxiety disorder pertains to an intense fear of being in one or more social situations where the person might feel embarrassed or humiliated because of potential judgment from others (Association, A. P, 2013). Individuals with social anxiety are more prone to encountering problems in their social relationships, experiencing feelings of loneliness, and facing difficulties with school activities or tasks (Russell and Topham, 2012; Vilaplana-Pérez et al., 2021). Problems in social relationships and feelings of loneliness lead individuals to restrict their social interactions, creating a vicious cycle that results in increased social stress and anxiety in the real world (Huan et al., 2014). Research indicates that individuals with social anxiety tend to use low-risk communication platforms (Ebeling-Witte et al., 2007), which can lead to problematic internet use (Campbell et al., 2006; Caplan, 2006; Liu and Kuo, 2007). In an example study, social anxiety fully mediated the relationship between loneliness and problematic internet use like excessive use of it over spending time in real life (Huan et al., 2014).

### 1.3 Present study-“the paradox of connection”

As previously discussed, humans have a fundamental need to establish and sustain meaningful and healthy social relationships. However, not all social interactions effectively fulfill this need. Different interpersonal relationship styles play a crucial role in individuals’ psychosocial well-being. Despite this, the existing literature has not sufficiently explored their relationship with social anxiety.

As previously mentioned, the nurturing relationship style supports mutual understanding and cooperation in interpersonal relationships and is associated with psychological well-being (Grenwald and Yıldırımoğlu, 1999). Researchers have found that individuals who grow up in a family environment characterized by nurturing and warmth during childhood tend to form more secure attachments in their close relationships, particularly in later life. This has been linked to the development of healthy emotion regulation mechanisms (Waldinger and Schulz, 2016). Individuals who adopt a nurturing relationship style may demonstrate more functional interpersonal behaviors in both in-person and online communication, and are likely to experience lower levels of social anxiety. In contrast, those with an inhibitory relationship style are expected to show lower functionality in these interactions.

As researchers have noted, while social interactions in the real world are declining, online communication is steadily increasing (Yao and Zhong, 2014). The rise of virtual relationships brings with it a variety of challenges. Although virtual communication has certain advantages, it can also give rise to difficulties that resemble or differ from those experienced in real-life relationships—such as exposure to cyberbullying, loneliness, and problematic internet use. For individuals who already experience social anxiety and interpersonal difficulties, the virtual world may offer new opportunities for communication but may also lead to adverse outcomes. In a recent systematic review, it was found that individuals with social anxiety tend to struggle with face-to-face interactions, while engaging more easily in online communication, seeking greater support through social media, and exhibiting more problematic patterns of internet use (O’Day and Heimberg, 2021). As individuals attempt to build relationships in virtual environments, they may paradoxically experience greater loneliness. Compared to in-person connections, virtual relationships are often weaker and more artificial, which may render them an illusion rather than a substitute for genuine social bonds.

In contemporary society, individuals increasingly seek meaningful social connections, yet often turn to digital platforms where interactions are typically more superficial and transient. Individuals with an inhibitory relationship style—characterized by emotional restraint and insecurity in attachment—may be particularly prone to initiating a pattern of social withdrawal, favoring digital communication over face-to-face interaction. For those experiencing social anxiety, this tendency is often reinforced, as the online environment presents a seemingly safer and more controllable alternative to in-person engagement. However, this shift to digital interaction, while initially reducing social discomfort, may paradoxically exacerbate feelings of loneliness over time, as virtual relationships often fail to fulfill deeper interpersonal and emotional needs. This dynamic reflects what we refer to in the present study as the *paradox of connection*—a phenomenon in which the pursuit of connection through digital means may ultimately intensify disconnection and perceived loneliness.

To this end, in this study, our goal was to examine the relationship between social anxiety and a previously unexplored concept—virtual environment loneliness—along with interpersonal relationship styles within a community sample. More specifically, we aim to investigate whether different dimensions of virtual environment loneliness, including virtual socialization, virtual sharing, and virtual loneliness, mediate the relationship between interpersonal relationship styles and social anxiety. Our hypotheses can be listed as follows:

*H*1: Nurturing relationship style is negatively associated with social anxiety.

*H*2: Inhibitory relationship style is positively associated with social anxiety.

*H*3: Virtual loneliness mediates the relationship between inhibitory relationship style and social anxiety.

## 2 Methods

### 2.1 Sample

The final sample of the study consisted of 400 adults (254 female, 146 male), ranging in age from 18 to 65 years residing in various regions of Turkey, including Kahramanmaraş, Osmaniye, İstanbul, Gaziantep, and Ankara. The survey sample consisted of 400 participants distributed across seven age groups. The largest proportion of respondents (48.3%) were aged 23–27, followed by 28–32 (19.5%) and 18–22 (14.0%). The least represented group was 43 and above (2.8%). Participants were recruited through social media platforms using a convenience sampling method. Inclusion criteria required participants to be between the ages of 18 and 65, fluent in Turkish, and capable of completing an online questionnaire independently. Individuals with self-reported diagnoses of severe psychiatric disorders (e.g., schizophrenia, bipolar disorder) were excluded to ensure data validity. Additionally, responses with extreme outliers or incomplete or inconsistent data were excluded from the final analysis.

The majority of participants were female (254; 63.5%) and held at least a bachelor’s degree (261; 65%). Regarding marital status, 65% of the participants were married, 32.8% were single, and 2% were divorced. This diverse sample allowed for a broader understanding of interpersonal relationship styles and social anxiety across a general adult population.

The procedures and purposes of the study were approved by the ethics committee of Hasan Kalyoncu University, with the decision date and number being 29.08.2024, E-97105791-050.04-64069.

### 2.2 Measures

Sociodemographic data form: A data form was administered to investigate participants’ age, gender, educational background, and marital status.

Liebowitz Social Anxiety Scale: Developed by Liebowitz in 1987, this scale is designed to measure the level of anxiety and avoidance experienced in performance-related situations and social environments (Liebowitz, 1987). The scale comprises 24 items rated on a 4-point Likert scale and includes two subdimensions: anxiety and avoidance. Sample items include *“Talking with people you do not know very well”* and *“Expressing a disagreement or disapproval to people you do not know very well.”* Each item is rated separately for the level of anxiety and the level of avoidance it elicits. The total score ranges from 0 to 144, with higher scores indicating greater social anxiety and avoidance. The adaptation of the scale into Turkish was conducted by Soykan et al. (2003). The Cronbach alpha score was 0.97 in this study.

Virtual Environment Loneliness Scale: The scale developed by Korkmaz and colleagues to assess the level of loneliness experienced by individuals in virtual environments, as well as their sharing and socialization, is a 5-point Likert type and consists of a total of 20 items (Korkmaz et al., 2014). It consists of three subdimensions: virtual socialization, virtual loneliness, and virtual sharing. In the Virtual Socialization subdimension, along with items such as “There are people in the virtual environment whom I feel close to,” there are also reverse-coded items like “I have no friends in the virtual environment.” In the Virtual Sharing subdimension, all items are positively coded, such as “I trust my virtual friends more than my real-life friends.” In the Virtual Loneliness subdimension, there are five reverse-coded items, including statements like “Friendships in virtual environments seem fake to me.” The Cronbach alpha score in this study was 0.80 for virtual socialization relationship style, 0.87 for virtual sharing, and 0.71 for virtual loneliness.

Interpersonal Relationships Scale: This scale was developed by Şahin and Durak (1994) to measure interpersonal relationship styles. The IRS consists of 31 items and uses a 4-point Likert scale. It has two subdimensions: nurturing and inhibiting. In the Nurturing Relationship subdimension, there are positive statements related to relationships, such as “I clearly show that I value the opinions and attitudes of others.” In contrast, the Inhibiting Relationship subdimension includes negative statements like “I insist on my own opinions and do not seek compromise.” High scores on the nurturing subdimension reflect a more positive relationship style, while high scores on the inhibiting subdimension indicate a more negative relationship style. The Cronbach alpha score in this study was 0.96 for nurturing relationship style and 0.95 for inhibitory relationship style.

### 2.3 Data analysis plan

Before carrying out the main analyses, several preliminary analyses were conducted. These included examining the scale characteristics, analyzing the intercorrelations among the study variables, and assessing the normality assumptions by evaluating skewness and kurtosis scores, where absolute values less than |2| were considered acceptable (Kline, 2023). Then, a series of mediation analyses were run. One of them included a parallel multi-mediation analysis so as to check whether virtual socialization, virtual sharing, and virtual loneliness mediate the association between inhibitory relationship style and social anxiety. After that, a second multi-mediation analysis replicated the same model except exchanging the exogenous variable of inhibitory relationship style with nurturing relationship style. All these two mediation analyses were performed by using the PROCESS macro (Model 4) for SPSS version 3.4 (Hayes, 2017). Additionally, internal consistency reliability analyses were conducted for all scales used in the study. The Cronbach’s alpha coefficients obtained were as follows: 0.97 for the Liebowitz Social Anxiety Scale, 0.80–0.87 for the subdimensions of the Virtual Environment Loneliness Scale, and 0.95–0.96 for the subdimensions of the Interpersonal Relationships Scale. These values indicate high internal consistency and are consistent with prior research, supporting the reliability of the measures used.

For assessing effect sizes, Cohen’s (1988) conventional thresholds were used, categorizing effect sizes as small (0.01–0.059), moderate (0.06–0.139), or large (≥0.14). In the mediation analyses, several key statistical indicators were reported to clarify the nature and strength of the observed effects. The total effect (c path) refers to the overall relationship between the independent variable (e.g., relationship style) and the dependent variable (e.g., social anxiety) without accounting for any mediators. The direct effect (c’ path) represents the effect of the independent variable on the dependent variable after controlling for the mediating variables. In contrast, the indirect effect (ab path) captures the portion of the relationship that is transmitted through one or more mediators (e.g., virtual socialization, virtual loneliness). To assess the explained variance in the dependent variable, R^2^ (squared multiple correlation) values were interpreted, with higher values indicating better model fit and greater predictive power. Furthermore, 95% confidence intervals (CI) for indirect effects were estimated using the bootstrap method with 5,000 resamples. When the confidence interval does not include zero, the mediation effect is considered statistically significant. This approach allows for robust estimation of mediation effects without relying on the assumption of normality (Hayes, 2017). All analyses were performed using SPSS (version 25).

## 3 Results

### 3.1 Preliminary analyses

Prior to testing the study’s main hypotheses, we analyzed the descriptive statistics, observed scale characteristics, normality assumptions, and correlations among the study variables. Initially, the normality assumptions of the variables were evaluated using skewness and kurtosis values. Within this scope, traditional threshold range between −2 and +2 was considered for deciding the assumption of normality. As displayed under Table 1, the skewness and kurtosis values for the current study variables ranged between 0.023 and 1.839, showing that the variables are normally distributed. All the descriptive analyses findings are listed under Table 1.

**Table 1:** Descriptive statistics results.

Variables	N	***X̄***	SD	Skewness	Kurtosis
Inhibitory relationship style	400	12.45	9.89	0.887	0.189
Nurturing relationship style	400	32.52	9.44	−1.134	1.839
Virtual socialization	400	23.39	6.34	0.023	−0.673
Virtual sharing	400	12.02	4.96	1.166	1.314
Virtual loneliness	400	16.44	4.06	0.153	−0.597
Social anxiety	400	100.58	30.83	0.239	−0.577

Preliminary analyses continued with the Pearson correlation analyses in respect to the main study variables. Regarding the results, first, inhibitory relationship style and nurturing relationship style were negatively correlated (*r* = −0.36, *p* < 0.001). While inhibitory relationship was positively related to social anxiety (*r* = 0.30, *p* < 0.001), nurturing one had a negative relationship with it (*r* = −0.21, *p* < 0.001). Considering the virtual environment loneliness subdimensions, social anxiety had only significant relationship with virtual sharing (*r* = −0.28, *p* < 0.001), but not the remaining subdimensions (*p* > 0.05).

Furthermore, inhibitory relationship style was significantly associated with virtual sharing (*r* = 0.20, *p* < 0.001), as well. On the other hand, nurturing relationship style was not significantly correlated with virtual sharing (*p* > 0.05), but it had significant associations with both virtual socialization (*r* = 0.27, *p* < 0.001) and virtual loneliness (*r* = −0.22, *p* < 0.001). Finally, some significant relationships were also observed among these virtual environment loneliness subdimensions. To illustrate, virtual socialization was positively related to both virtual sharing (*r* = 0.32, *p* < 0.001) and virtual loneliness (*r* = 0.14, *p* < 0.001). However, no significant relationship was captured between virtual sharing and virtual loneliness (*p* > 0.05). All these correlational findings were displayed under Table 2.

**Table 2:** Correlation analysis findings (*N* = 400).

	1	2	3	4	5	6
1. Inhibitory Relationship Style	-					
2. Nurturing Relationship Style	−0.36**	-				
3. Virtual Socialization	−0.04	0.27**	-			
4. Virtual Sharing	0.20**	0.04	0.32**	-		
5. Virtual Loneliness	−0.03	−0.22**	0.14**	−0.05	-	
6. Social Anxiety	0.30**	−0.21**	−0.06	0.28**	−0.07	-

**Correlation is significant at 0.01 level. *Correlation is significant at 0.05 level.

### 3.2 Mediation analyses

**Figure 1:**
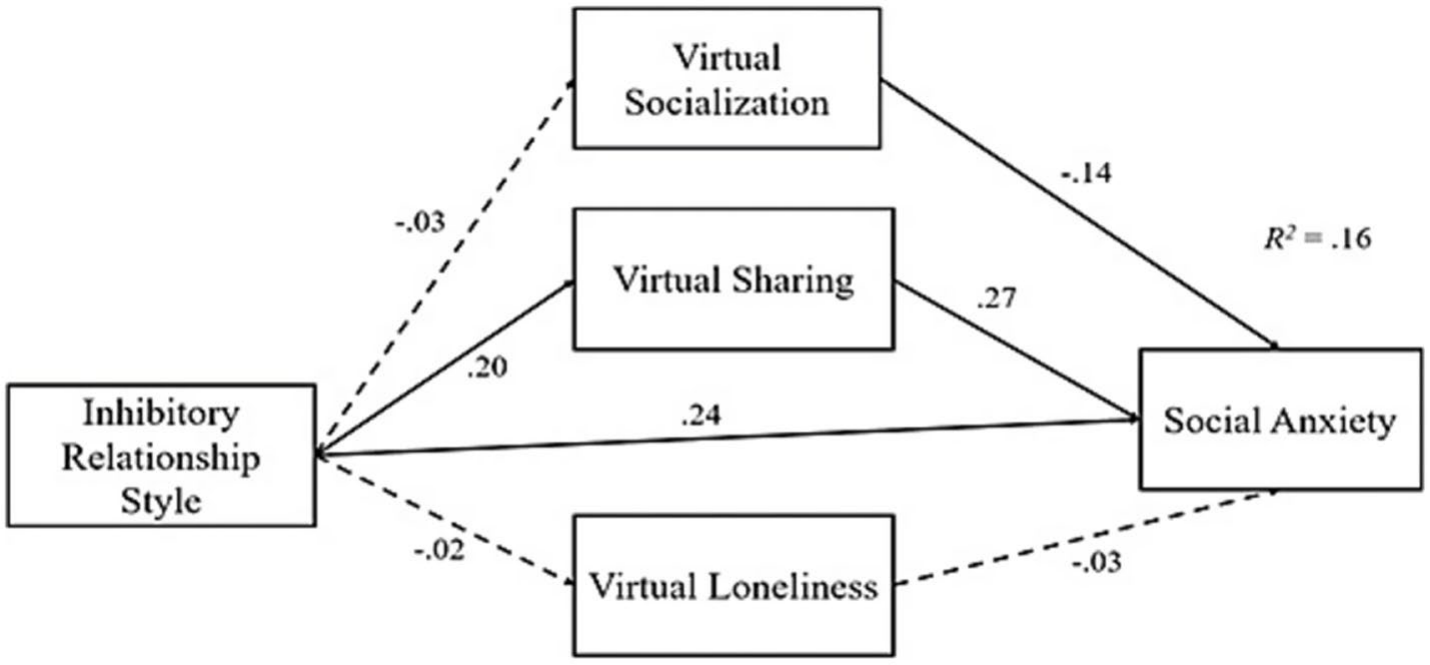
Standardized regression effects of the first mediation analysis.

In order to examine the potential mediating roles of virtual socialization, virtual sharing, and virtual loneliness in the relationship between the two relationship styles (inhibitory vs. nurturing) and social anxiety, two parallel multi-mediation models were tested for each relationship styles. First mediation analysis results regarding the inhibitory relationship style revealed that it significantly predicted virtual sharing (*β* = 0.20, *p* < 0.001) and social anxiety (*β* = 0.24, *p* < 0.001), but not virtual socialization and virtual loneliness (*p* > 0.05). Moreover, along with inhibitory relationship style, both virtual sharing (*β* = 0.27, *p* < 0.001) and virtual socialization (*β* = −0.14, *p* < 0.01) emerged as significant predictors of social anxiety. Moving to the indirect effects, solely virtual sharing mediated the relationship between inhibitory relationship style and social anxiety (*β* = 0.05, 95% CI = 0.023, 0.089). That is to say that having inhibitory relationship style is related to increased virtual sharing, which in return is correlated with higher social anxiety. All these results are provided under Table 3 and Figure 1.

**Table 3:** Standardized coefficients for the first mediation model (Inhibitory relationship style).

Predictor	Outcome											
	*M_1_*			*M_2_*			*M_3_*			*Y*		
	*Coeff.*	*SE*	*p*	*Coeff.*	*SE*	*p*	*Coeff.*	*SE*	*p*	*Coeff.*	*SE*	*p*
X (Inhibitory Rel. S.)	−0.03	0.03	0.477	0.20	0.02	0.000	−0.03	0.02	0.590	0.24	0.14	0.000
*M_1_* (V. Socialization)	-	-	-	-	-	-	-	-	-	−0.14	0.24	0.006
*M_2_* (V. Sharing)	-	-	-	-	-	-	-	-	-	0.27	0.31	0.000
*M_3_* (V. Loneliness)	-	-	-	-	-	-	-	-		−0.03	0.35	0.505
Constant	23.67	0.51	0.477	10.79	0.39	0.000	16.58	0.32	0.000	90.22	7.97	0.000
	*R^2^* = 0.00; *p = 0.47*			*R^2^* = 0.04; *p* < 0.01			*R^2^* = 0.00; *p = 0*.59			*R^2^* = 0.16; *p* < 0.01		
Indirect effect between inhibitory relationship style and social anxiety through virtual sharing												
*Coeff.*		Boot SE				Boot LLCI 95%			Boot ULCI 95%			
0.05		0.01				0.02			0.09			

Rel., Relationship; S., Style; V, Virtual.

**Figure 2:**
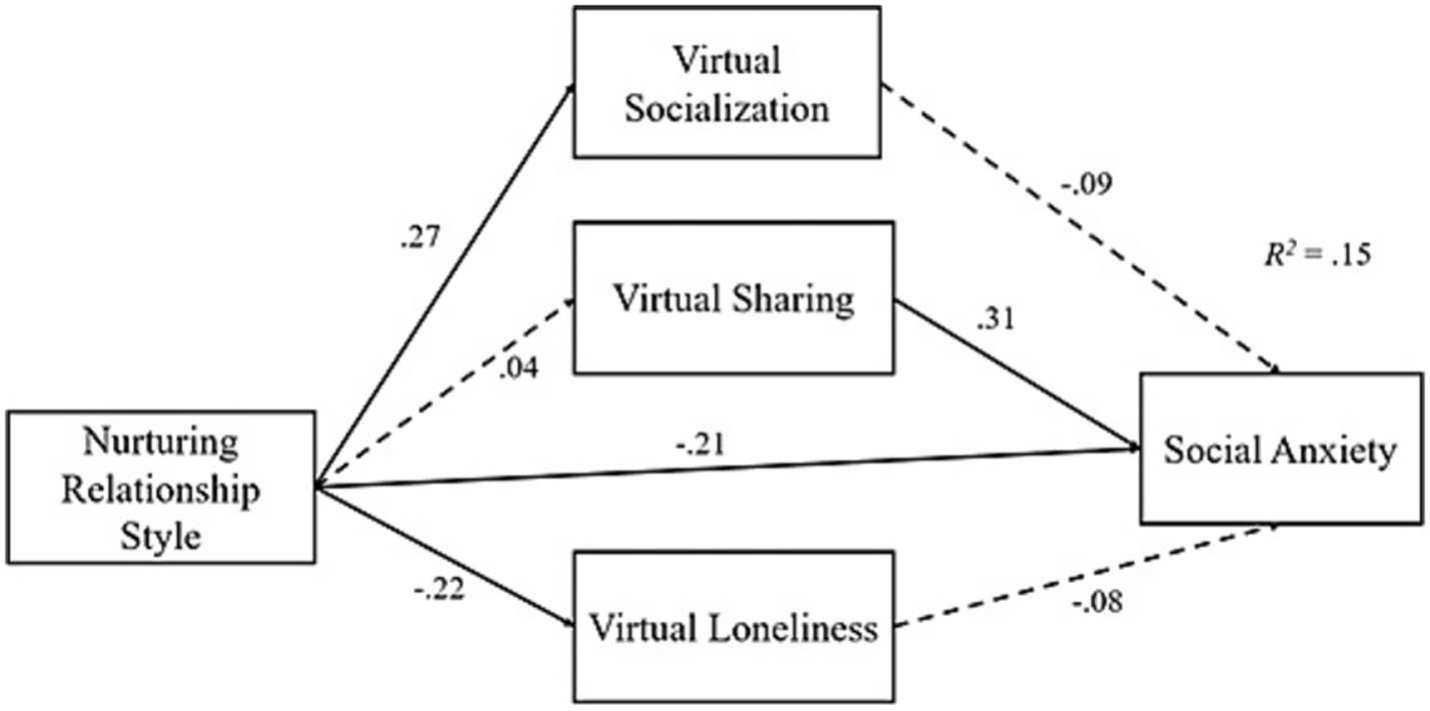
Standardized regression effects of the second mediation analysis.

Same mediation analysis was replicated by replacing inhibitory relationship style with nurturing relationship style. This second round mediation analysis results showed that nurturing relationship style positively predicted virtual socialization (*β* = 0.27, *p* < 0.001), but it negatively predicted virtual loneliness (*β* = −0.22, *p* < 0.001) and social anxiety (*β* = −0.21, *p* < 0.001), but not virtual sharing (*p* > 0.05). In addition, virtual sharing was the only significant predictor of social anxiety among the virtual environment loneliness indicators (*β* = 0.31, *p* < 0.001). In terms of the indirect effects, none of hypotheses were supported. All these findings are displayed under Table 4 and Figure 2.

**Table 4:** Standardized coefficients for the second mediation model (Nurturing relationship style).

Predictor	Outcome											
	*M_1_*			*M_2_*			*M_3_*			*Y*		
	*Coeff.*	*SE*	*p*	*Coeff.*	*SE*	*p*	*Coeff.*	*SE*	*p*	*Coeff.*	*SE*	*p*
X (Nurturing Rel. S.)	0.27	0.03	0.00	0.04	0.02	0.449	−0.22	0.02	0.000	−0.21	0.16	0.000
*M_1_* (V. Socialization)	-	-	-	-	-	-	-	-	-	−0.09	0.25	0.082
*M_2_* (V. Sharing)	-	-	-	-	-	-	-	-	-	0.31	0.30	0.000
*M_3_* (V. Loneliness)	-	-	-	-	-	-	-	-		−0.09	0.37	0.070
Constant	17.48	1.09	0.000	11.37	0.89	0.000	19.55	0.71	0.000	121.49	9.59	0.000
	*R^2^* = 0.07; *p* < 0.01			*R^2^* = 0.00; *p = 0*.44			*R^2^* = 0.05; *p* < 0.01			*R^2^* = 0.14; *p* < 0.01		

No indirect effect was captured. Rel., Relationship; S., Style; V, Virtual.

## 4 Discussion

The aim of this study was to examine the relationships between social anxiety, virtual environment loneliness, and interpersonal relationship styles. The sample encompassed a wide age range, from adolescents to older adults, with the majority of participants being female (63.5%) and having a high level of education (65%). This context is significant for understanding the effects on social interactions. Women are known to be more sensitive in social relationships and more active in seeking social support (Taylor et al., 2000). Additionally, individuals with higher education levels typically have better access to resources and information, enabling them to manage stress more effectively. This context allows for a broader perspective on the evaluation of the study’s results.

The findings indicated a positive correlation between the inhibitory relationship style and social anxiety, while the nurturing relationship style showed a negative correlation with it. This supports the idea related to close connection between social anxiety and inhibitory relationship style. That is to say that having negative interpersonal relationship style is related to adverse mental health outcomes, as also highlighted by previous research (Şahin et al., 2011b; Doğan and Sapmaz, 2012).

From a theoretical standpoint, the association between inhibitory relationship style and social anxiety can be interpreted through the lens of Attachment Theory (Bowlby, 1969) and the Cognitive-Behavioral Model of social anxiety (Bowlby, 1969; Clark and Wells, 1995). According to Bowlby, early interactions with caregivers shape internal working models of the self and others, influencing later interpersonal behaviors and emotional regulation. Individuals with an inhibitory relationship style often exhibit patterns consistent with insecure attachment, particularly avoidant attachment, characterized by discomfort with closeness and emotional intimacy. These early attachment insecurities contribute to the development of negative self-beliefs, heightened sensitivity to social evaluation, and hypervigilance to rejection—all central components in Clark and Well (1995) cognitive-behavioral model of social anxiety.

Inhibitory individuals tend to expect rejection or criticism, leading them to avoid emotional disclosure and suppress expressions of vulnerability. This avoidance limits opportunities to build trust and receive positive social feedback, thus perpetuating feelings of isolation and anxiety in social contexts. The interplay between these cognitive and attachment-related factors creates a feedback loop that sustains social anxiety symptoms, as individuals may continue to withdraw to protect themselves from anticipated social threat.

Inhibitory style appears to damage health acquisition of relatedness need and social support, and may lead to adverse outcomes like social anxiety. This style may encourage avoidance behaviors and emotional withdrawal, limiting opportunities for positive social feedback and secure attachments. Consequently, a vicious cycle can develop between this dysfunctional relationship style and mental health problems. Then, even it might form a vicious circle between this dysfunctional relationship style and mental health problems. For instance, Russell and Topham (2012) noted that individuals with social anxiety face difficulties in social relationships, which can increase emotional isolation (Russell and Topham, 2012). Inhibitory individuals may rely more heavily on virtual interactions as a compensatory strategy, but these interactions often lack the richness and emotional attunement needed to satisfy fundamental social needs, thus potentially exacerbating anxiety symptoms. Conversely, the negative correlation between the nurturing relationship style and social anxiety relates to individuals’ ability to seek social support and cope with stress (Cohen and Wills, 1985).

Our mediation analyses highlighted the significant mediating role of specific virtual environment loneliness dimension, namely virtual sharing, in the relationship between inhibitory relationship style and social anxiety. The results showed that increase in the inhibitory relationship style was associated with greater virtual sharing, which, in turn, heightened social anxiety. This finding aligns with the work of Huan et al. (2014), suggesting that social anxiety can limit individuals’ interactions in virtual environments, potentially leading to problematic internet use (Huan et al., 2014). Thus, individuals with an inhibitory relationship style may engage in more sharing in virtual spaces as a form of “digital escape,” which can exacerbate their social anxiety. This finding supports existing literature emphasizing the close link between interactions in virtual settings and social anxiety (Caplan, 2006). We elaborate that this result might reflect a maladaptive compensatory strategy. Individuals with an inhibitory style may avoid in-person social risks and instead rely on virtual spaces, which lack the nonverbal feedback and emotional attunement necessary for building secure social bonds. This overreliance on digital interaction may paradoxically intensify social anxiety, as it perpetuates avoidance and fails to provide corrective interpersonal experiences.

Although the nurturing style was associated with lower social anxiety and increased virtual socialization, it did not exert a significant mediation effect via virtual sharing. One possible explanation is that individuals with a nurturing style derive emotional security primarily from direct social interactions rather than virtual sharing, which may be perceived as superficial or less fulfilling. Therefore, their social anxiety may be more directly alleviated through high-quality offline relationships, reducing the relevance of virtual sharing as a mediating pathway.

On the other hand, the nurturing relationship style was found to increase virtual socialization while decreasing virtual loneliness, demonstrating its positive impact on forming social connections. The increase in virtual socialization helps alleviate feelings of loneliness, thereby reducing levels of social anxiety. Morahan-Martin and Schumacher (2003) point out that virtual interactions offer opportunities for developing healthy social connections, suggesting that individuals with a nurturing relationship style are more likely to establish meaningful interactions in virtual environments (Morahan-Martin and Schumacher, 2003).

The mediating role of virtual sharing in the relationships between inhibitory relationship style and social anxiety underscores the critical importance of managing virtual interactions and developing healthy social connections for individuals with social anxiety. Future research could further explore the role of virtual environment loneliness in these dynamics, contributing to the development of more effective coping strategies for social anxiety.

In conclusion, this study provides important insights into the complex interactions between social anxiety, virtual loneliness, and interpersonal relationship styles, while emphasizing the mediating effect of virtual sharing in these relationships. Effectively managing social interactions is crucial for enhancing individuals’ psychological well-being.

In light of our findings, intervention efforts targeting individuals with inhibitory relationship styles might benefit from approaches such as social skills training, assertiveness training, and attachment-based group therapies (Spence, 2003; Mikulincer and Shaver, 2010; Speed et al., 2018). These interventions can help reduce avoidance behaviors, foster secure emotional expression, and promote healthier patterns of social engagement. Emotion regulation training, particularly in recognizing and expressing interpersonal needs, may also buffer against the development or worsening of social anxiety symptoms (Berking et al., 2008).

### 4.1 Limitations

Despite the contributions of the current findings, the present study is not without limitations. First, the cross-sectional design prevents any causal inferences regarding the relationships between interpersonal relationship styles, virtual environment loneliness, and social anxiety. Future research would benefit from longitudinal or experimental designs to establish the directionality of these associations. Second, the study relies on self-report measures, which may be subject to social desirability bias or inaccuracies in participants’ self-perceptions. Incorporating behavioral assessments or multi-informant reports could enhance the validity of findings. Third, although the sample includes participants from various age groups and regions, it is not fully representative, as approximately two-thirds of the participants were women and the age distribution was broad. We did not statistically control for gender or age in the current mediation models, which may have influenced the findings. Future studies should consider including these variables as covariates to better understand their potential effects. Finally, while the sample provides valuable insights, its generalizability may still be limited. Future studies should aim for more diverse and representative samples in terms of age, gender, cultural background, and digital engagement levels.

## Data Availability

The original contributions presented in the study are included in the article/Supplementary material, further inquiries can be directed to the corresponding author.
